# Successful Surgical Management of a Giant Cell Tumor in the Proximal Tibia: A Case Report

**DOI:** 10.7759/cureus.59173

**Published:** 2024-04-27

**Authors:** Milind R Gharpinde, Aditya Pundkar, Sandeep Shrivastava, Rohan Chandanwale, Hardik Patel

**Affiliations:** 1 Orthopedics, Jawaharlal Nehru Medical College, Datta Meghe Institute of Higher Education and Research, Wardha, IND

**Keywords:** rehabilitation, autologous fibula graft, preoperative embolization, surgical management, proximal tibia, giant cell tumor

## Abstract

Giant cell tumors (GCTs) of the bone present unique challenges in management due to their locally aggressive nature and potential for recurrence. This case report describes the successful surgical management of a GCT located in the proximal tibia of a 28-year-old female. The patient presented with six months of pain and swelling following a traumatic injury to the knee. Diagnostic imaging confirmed the presence of a GCT, leading to preoperative prophylactic embolization to reduce intraoperative bleeding. Surgical excision of the tumor was performed, followed by reconstruction using autologous fibula grafts and plate fixation. Postoperative care included analgesia, antibiotics, and physiotherapy. Regular follow-up demonstrated satisfactory clinical outcomes without evidence of recurrence. This case highlights the importance of a multidisciplinary approach combining surgical expertise, preoperative planning, and postoperative rehabilitation to achieve favorable outcomes in managing GCTs.

## Introduction

Giant cell tumors (GCTs) of bone are uncommon but locally aggressive neoplasms, constituting approximately 5% of all primary bone tumors [1]. They typically arise in the epiphyses of long bones, with a predilection for the distal femur, proximal tibia, and distal radius [2]. GCTs most commonly affect individuals aged 20 to 40 years, with a slight predilection for females [3]. Histologically, GCTs are characterized by multinucleated giant cells interspersed among mononuclear stromal cells [4]. While benign, GCTs exhibit locally aggressive behavior, with a propensity for recurrence if inadequately treated [5]. The mainstay of treatment for GCTs involves surgical resection, aiming for complete excision while preserving joint function and minimizing morbidity [6].

Despite advances in surgical techniques, managing GCTs in weight-bearing bones such as the proximal tibia poses challenges due to the risk of structural instability and compromised limb function postresection [7]. Additionally, intraoperative bleeding during tumor excision can be significant, necessitating strategies such as preoperative embolization to minimize blood loss [8]. This case report highlights the successful surgical management of a GCT in the proximal tibia of a young female patient, emphasizing the importance of a multidisciplinary approach incorporating preoperative embolization, meticulous surgical technique, and postoperative rehabilitation.

## Case presentation

**Figure 1:**
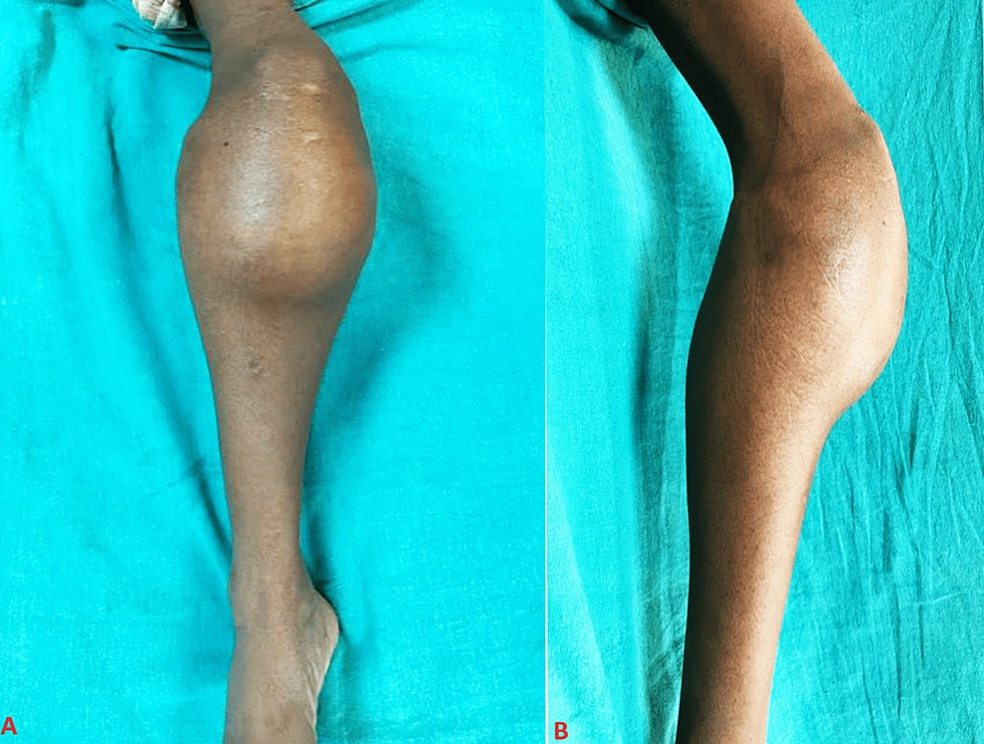
(A-B) Swelling over the right knee and the proximal tibia measuring approx. 15 x 12 cm

A 28-year-old female presented to the Orthopedic Outpatient Department with complaints of persistent pain and swelling over her right knee, persisting for six months. The symptoms initiated abruptly following a fall from a moving car, where she sustained an injury to her right knee. She described the pain as severe, constant, nonprogressive, and exacerbated by movement but relieved by rest. The physical examination revealed a significant swelling measuring approximately 15 x 12 cm over the proximal tibia and tense and reddened skin over the affected area (Figure 1).

**Figure 2:**
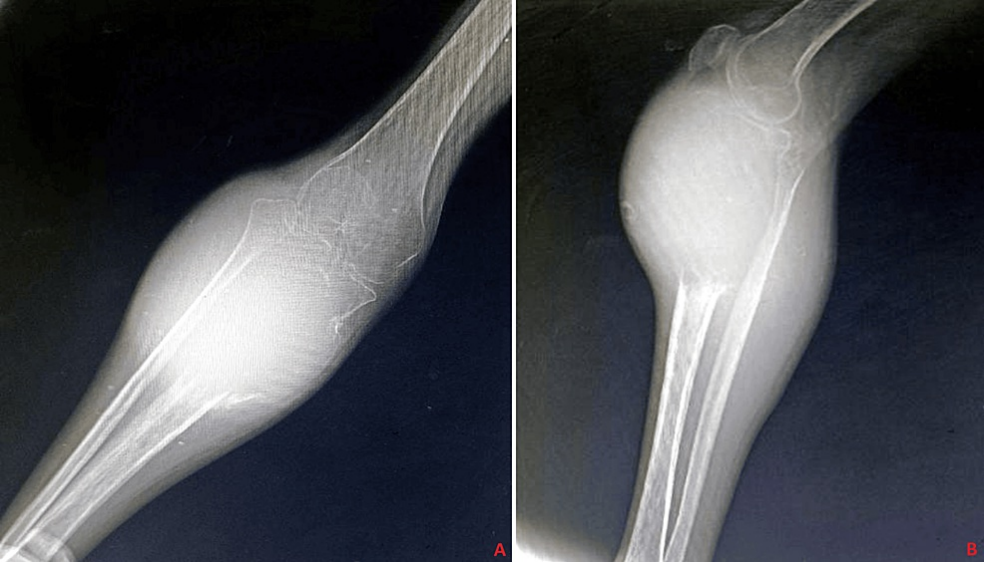
(A-B) X-ray of the right knee with tibia suggestive of osteosclerotic lesion with ill-defined margin over the proximal tibia and periosteal reaction present

Given the clinical presentation, the attending surgeon admitted the patient for further evaluation. Diagnostic imaging included X-rays of the right knee in anteroposterior and lateral views, which revealed a lytic lesion suggestive of a GCT in the proximal tibia (Figure 2). The decision to perform preoperative prophylactic embolization was made to reduce intraoperative blood loss. This procedure involved accessing the right femoral artery to perform an angiogram, identifying tumor-feeding arteries from the lateral genicular branch of the popliteal artery, a synovial branch of the popliteal artery, and a branch of the fibular artery. Selective cannulation of these feeders was achieved using a microcatheter, followed by embolization with microparticles to occlude blood flow to the tumor.

**Figure 3:**
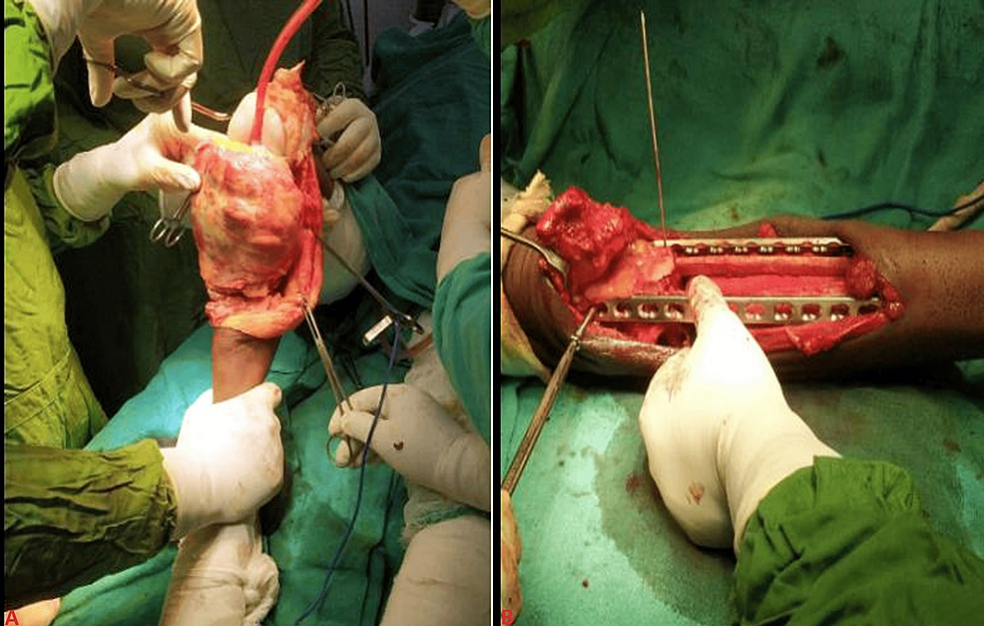
Intraoperative procedure of bone tumor excision and limited contact dynamic compression plating

Following thorough counseling and obtaining informed consent from the patient and her family, surgical intervention was carried out under spinal anesthesia. The surgery involved the complete excision of the tumor along with the surrounding soft tissue. To manage the resultant defect, a segment of the distal femoral condyle was resected, and the medially opposite site fibula was harvested and inserted proximally into the femoral medullary cavity and distally into the tibial medullary cavity. This construct was stabilized using a 16-hole limited-contact dynamic-compression plate (LCDCP). Additionally, the fibula was prepared and tibialized and then fixed with an 18-hole LCDCP (Figure 3). Small bone grafts from the distal femoral condyles were placed on the eroded surfaces of the fibular grafts to promote integration and stability. Platelet-rich plasma (PRP) infiltration was also administered to enhance healing.

**Figure 4:**
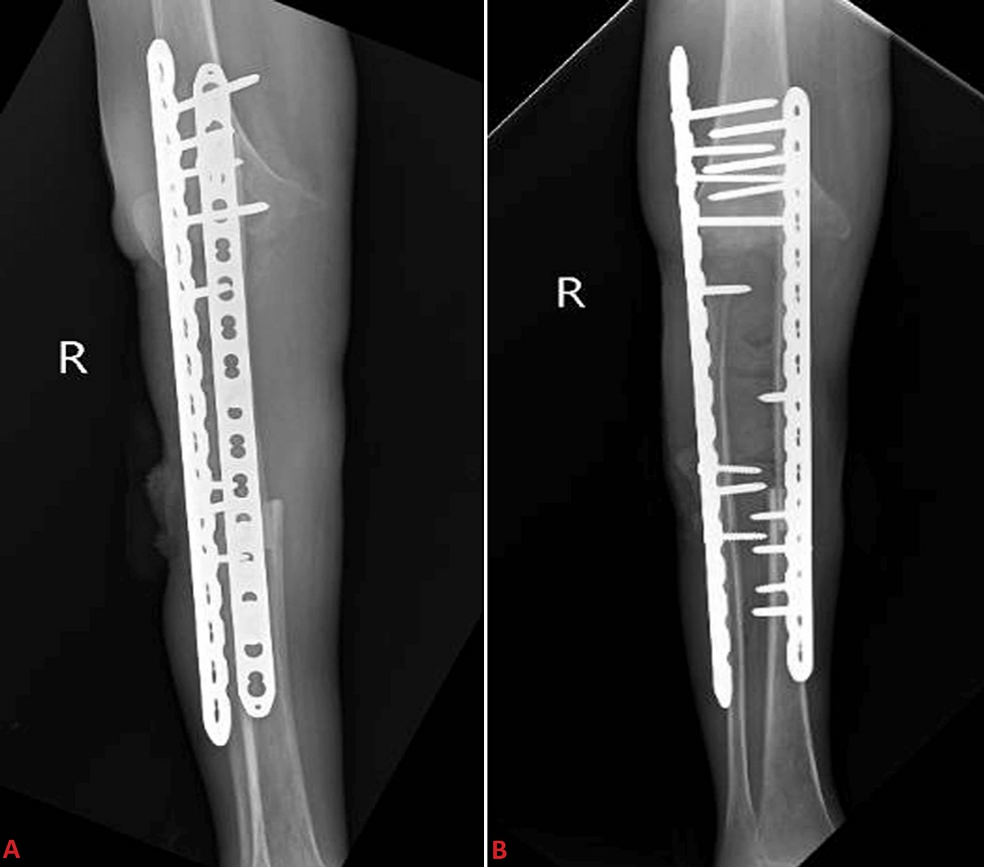
Postop X-ray of the right knee with leg

Postoperatively, the patient received a comprehensive care, including pain management with analgesics, infection prevention with antibiotics, and antacid administration to counteract medication side effects. The wound care involved regular sterile dressings to promote healthy granulation tissue at the surgical site. Rehabilitation started early with physiotherapy focusing on strengthening exercises for the quadriceps and hamstrings and straight leg raises to facilitate full weight-bearing mobilization using a walker. Postoperative X-rays are shown in Figure 4.

**Figure 5:**
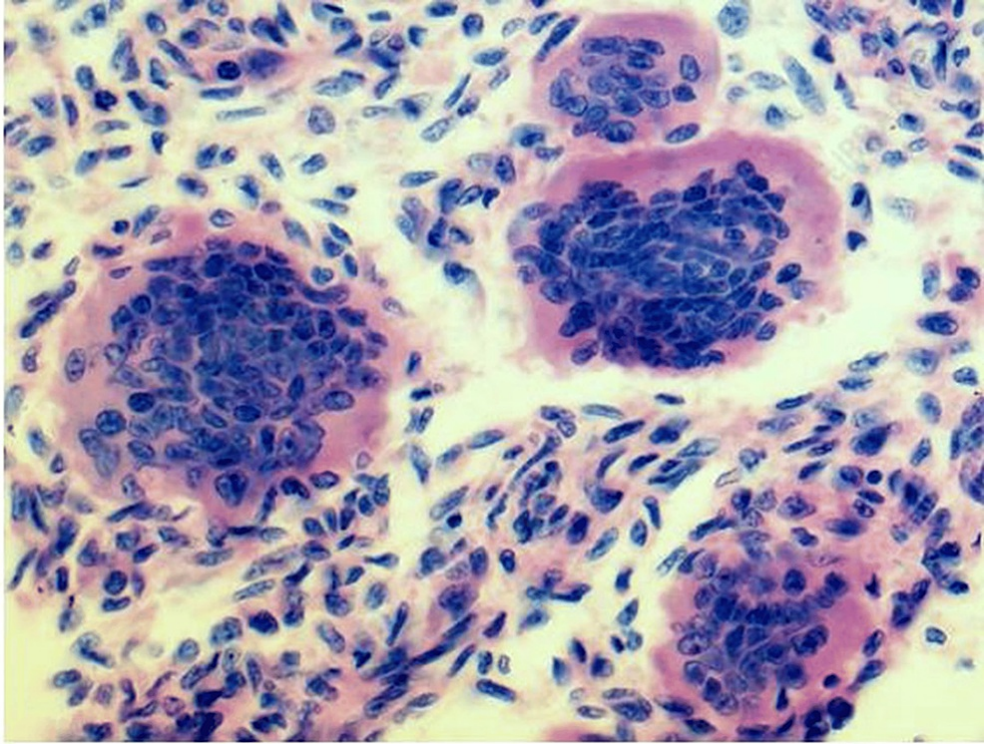
Giant cell tumor

Histopathological analysis of the resected bone confirmed the diagnosis of a GCT (Figure 5). The patient was discharged in a stable condition with intact distal circulation and was advised to continue oral medications and regular follow-up. Six and 12-month follow-up visits showed no signs of recurrence on imaging, and the patient reported satisfactory functional recovery, achieving full weight-bearing capacity without discomfort.

## Discussion

The successful management of GCTs in weight-bearing bones poses significant challenges due to their aggressive nature and potential for local recurrence. This case report illustrates a comprehensive approach to the surgical management of a GCT located in the proximal tibia of a 28-year-old female. The integrated strategy involved preoperative embolization to minimize intraoperative blood loss, meticulous surgical resection, and autologous fibula graft reconstruction. Preoperative embolization is a well-established technique to reduce blood loss during surgery for highly vascular tumors, such as GCTs [9]. In this case, selective embolization of tumor-feeding arteries from the lateral genicular and synovial branches of the popliteal artery and a branch of the fibular artery was performed successfully. This approach has decreased intraoperative blood loss and transfusion requirements, facilitating a safer surgical excision [10].

Surgical resection remains the mainstay of treatment for GCTs, aiming for complete tumor removal while preserving joint function and minimizing the risk of recurrence [7]. In our case, a wide excision of the tumor and a segment of the distal femoral condyle were performed to ensure adequate margins. Reconstruction of the bone defect was achieved using autologous fibula grafts, which provide structural support and promote osseous integration [11]. The stabilization of the fibula and fixation with LCDCPs allowed for stable alignment and early mobilization, which is crucial for functional recovery. PRP infiltration in conjunction with surgical reconstruction has gained popularity recently for its potential to enhance bone healing and reduce complications [12]. In our case, PRP was administered intraoperatively to augment the healing process and promote bone union. While the exact mechanisms of PRP's action remain under investigation, its application in orthopedic surgery, particularly in complex reconstructions, shows promising results.

Follow-up evaluation is essential for monitoring postoperative outcomes and detecting recurrence early. Imaging modalities such as X-rays and MRI are crucial in assessing bone healing and detecting any signs of tumor recurrence [12]. In this case, regular follow-up visits at six and 12 months postsurgery demonstrated satisfactory clinical outcomes with no evidence of recurrence on imaging.

## Conclusions

In conclusion, the successful management of GCTs in weight-bearing bones demands a comprehensive approach integrating preoperative embolization, meticulous surgical excision, and effective postoperative rehabilitation. This case report exemplifies the efficacy of such an approach in treating a GCT located in the proximal tibia of a 28-year-old female. By employing preoperative embolization to minimize intraoperative blood loss and utilizing autologous fibula grafts for reconstruction, along with adjunctive measures like PRP infiltration, favorable outcomes were achieved. Regular follow-up evaluations demonstrated satisfactory clinical results with no evidence of tumor recurrence. This case underscores the importance of a multidisciplinary approach. It highlights the potential for successful outcomes in managing complex bone tumors like GCTs when guided by evidence-based principles and integrated care pathways.
